# Mechanical and microstructural assessment of conventional carbon and stainless steel shear stud welded connections

**DOI:** 10.1038/s41598-026-37051-7

**Published:** 2026-02-03

**Authors:** Hizb Ullah Sajid, Ryan Slein

**Affiliations:** 1 Turner-Fairbank Highway Research Center, Genex Systems LLC, McLean, VA 22101 USA; 2 Turner-Fairbank Highway Research Center, Federal Highway Administration, McLean, VA 22101 USA

**Keywords:** Shear studs, Stainless steel, Arc stud welding, Mechanical properties, Weld microstructure, Weld microhardness, Engineering, Materials science

## Abstract

Shear studs are crucial for composite action between a bridge’s superstructure and deck. While carbon steel shear studs weld well onto ASTM A709 Grade 50 steel, their use with weathering bridge steels, particularly ASTM A709 Grade 50CR, has been associated in the literature with potential service-life concerns such as galvanic corrosion arising from metallic dissimilarities. Austenitic stainless steel shear studs have therefore been proposed for use with martensitic ASTM A709 Grade 50CR; however, literature on their mechanical behavior is limited, and code guidance is lacking. This study aims to investigate the mechanical performance and failure mechanisms in conventional carbon and austenitic stainless steel shear studs welded to ASTM A709 Grade 50 and Grade 50CR base metals. Shear and tensile tests were performed on three stud–base metal configurations: (1) a carbon steel shear stud welded to an ASTM A709 Grade 50 plate, (2) a carbon steel shear stud welded to an ASTM A709 Grade 50CR plate, and (3) an austenitic stainless steel shear stud welded to an ASTM A709 Grade 50CR plate. Weld-zone microstructural analysis and microhardness traverses were conducted for each stud–plate assembly. Tensile and shear testing demonstrated an increase in tensile and shear capacities and an increase in ductility for austenitic studs compared with carbon steel studs. Microstructural analysis revealed martensite formation in the coarse-grained heat-affected zone (CGHAZ) of the carbon steel stud welds with Vickers hardness measurements reaching 390 HV0.5. The stainless steel shear stud welds consistently reached 430 HV0.5 in the CGHAZ and weld microstructure, which may be attributed to the formations of martensite, secondary phases, and carbides in these regions. These elevated hardness levels may affect local toughness and increase susceptibility to cold cracking. This significant hardness increase underscores the need to maintain pre‑qualified stud‑welding parameters including heat input, correct stud seating and hold, and surface cleanliness, to ensure consistent weld reliability. The results obtained from this study aim to provide bridge owners valuable insights into the performance and reliability of stainless steel shear studs for bridge applications.

## Introduction

Steel shear studs are widely used in composite bridge construction to transfer shear forces between the concrete deck and steel girders. Typically made from low-carbon steel grades, these studs are welded onto steel girders via arc stud welding techniques^1,2^. The mechanical properties of steel shear studs are crucial for their role in ensuring composite action in bridges. Those properties are determined by stud geometry, material properties, and weld quality^3,4^. The number and spacing of shear studs on steel girders are guided by the AASHTO’s *LRFD Bridge Design Specifications*, with minimum spacing at least six times the diameter of the stud^5^. The chemical properties of the studs generally align with the specifications of ASTM A709^6^ steel girders.

In areas with nominal‑to‑high atmospheric corrosion, weathering steels such as ASTM A709 Grade 50 W reduce maintenance and coating needs; however, their protective patina can fail in chloride‑rich or persistently wet environments, leading to premature corrosion and repair needs^7–10^. Studies of bridges in Texas^9^ and Iowa^10^ revealed patina failure and delamination on Grade 50 W steel, allowing chloride and moisture penetration that caused corrosion and early repairs. These field observations prompted FHWA Technical Advisory 5140.22 and further guidance following the 2022 Pittsburgh bridge investigation^11–16^. Consequently, interest has grown in corrosion‑resistant alternatives such as ASTM A1010 (now A709 Grade 50CR), which motivated the material choices examined in this study.

Extensive research has focused on mitigating corrosion, which has resulted in the development of coatings, bio-based corrosion inhibitors and deicers, new corrosion-resistant steel grades, and more. Among them, the use of corrosion-resistant steel in bridge construction has become an attractive option for reducing long-term maintenance costs. Various stainless steel grades have been researched, with their performance in different corrosive environments well documented in the literature^17–19^. Notably, ASTM A709 Grade 50CR, also known as ASTM A1010, has gained widespread attention for its cost-effectiveness and excellent corrosion resistance in bridge applications. This low-alloy stainless steel, which contains 10 to 12.5% chromium, has been studied extensively in recent years and has been used in the construction of several bridges across the United States^20–26^. In addition, studies have suggested that ASTM A1010 is more economical through the course of a service life of more than 20 years compared with coated 50 W steel^18^. Due to its advantageous properties, in 2017, ASTM A1010^27^ became incorporated into the ASTM A709 specifications^6^ as Grade 50CR steel.

Despite Grade 50CR’s adoption, conventional carbon steel studs remain commonly used for fabricating shear studs in composite bridges. While carbon steel shear studs are compatible with ASTM A709 Grade 50 W, literature has reported that welding them onto Grade 50CR can introduce material mismatches, which may lead to potential galvanic corrosion. Previous studies have also shown that significant differences in the chemical composition, crystal structure, and thermal expansion properties between base metals and weld metals create complexities in the fusion zone microstructure^28–31^, which can adversely influence the mechanical performance and corrosion resistance of dissimilar weld fusion zones^32–35^.

Prior work on dissimilar metal welds (DMWs) has shown that microstructure and chemical gradients influence electrochemical behavior and local corrosion susceptibility—for example, broader martensitic layers correlated with lower Volta potential and galvanic activity at ferritic–nickel interfaces^36^, and coarse-grained HAZ regions were found particularly vulnerable under aggressive conditions^37^. To avoid those complications, stainless steel shear studs have been proposed for use with stainless steel-grade base plates; however, literature on their mechanical behavior is limited, as is guidance on AASHTO and AWS codes. While many DMW studies examine varied alloy systems and corrosion mechanisms (including galvanic -and stress-assisted corrosion)^29,36,37^, targeted investigations of Grade 50CR fusion with mild or austenitic stainless studs—and their mechanical behavior under studweld conditions—are limited.

To fill these gaps, this study presents the first systematic experimental comparison of carbon versus austenitic stainless shear studs welded to ASTM A709 Grade 50 and Grade 50CR (A1010) base plates. We conducted shear and tensile tests to determine ultimate loads, elongation, and failure modes, and carried out detailed weld-zone microstructural examinations and microhardness traverses to link mechanical behavior with local microstructural changes. The results provide quantitative data and mechanistic insight to support informed use of stainless studs in bridge applications and to guide future corrosion and code-development work.

## Experimental procedure

This section outlines tensile and shear testing protocols, failure mode evaluations, microhardness indentation transverse across welds, and optical microscopy conducted for the weld zones. The methods are applicable to mild steel and stainless steel studs and substrates.

### Materials

To fabricate steel shear stud specimens, mild steel plates conforming to ASTM A709 Grade 50^6^ and Grade C-1015 mild steel shear studs conforming to ASTM A108^38^ and AWS D1.1^39^ requirements were obtained from a commercial supplier. Similarly, A1010^6^ steel plates and 316L stainless steel shear studs that conform to ASTM A276^40^ and AWS D1.6^41^ requirements were obtained. The A1010 plate was made from a heat prior to the adoption of Grade 50CR into ASTM A709^33^; however, the plate still meets chemical compliance. The mechanical and chemical properties of these steel grades, obtained from supplied-provided certificates and in-house tests, are provided in Tables 1 and 2, respectively. The studs used are in compliance with AWS D1.5 Table 9.1^42^ and AASHTO LRFD Article 6.4.4^5^ that require welded stud shear connectors to have a minimum specified yield of 50 ksi when used for transfer of shear in a composite system.

The steels selected reflect common practice in composite bridge construction. ASTM A709 Grade 50 mild steel plates and Grade C-1015 mild studs (ASTM A108; AWS D1.1/D1.5) were chosen because Grade 50 studs are the standard connectors specified by AASHTO LRFD Article 6.4.4 for composite shear transfer. A1010 stainless steel plates (now ASTM A709 Grade 50CR) and 316Lstainless steel studs (ASTM A276; AWS D1.6) were included to evaluate corrosion-resistant systems increasingly used in aggressive environments and to address gaps in mechanical and weld-zone behavior data for such combinations. Together, these materials enable a direct comparison between conventional and corrosion-resistant stud–plate systems relevant to current bridge practice.

**Table 1:** Mechanical properties of the steel grades.

Steel type	Yield strength, ksi	Ultimate strength, ksi	Percent elongation
C-1015 mild steel stud	51.1	69.1	29.0 (2-in. gauge length)
Grade 50CR base plate	50.2	75.0	47.0 (2-in. gauge length)
Grade 50 base plate	56.4	72.9	25.0 (8-in. gauge length)
316L stainless steel stud	48.5	83.9	57.0 (2-in. gauge length)

**Table 2:** Elemental composition of the steel grades.

Steel type	C	Mn	Si	*P*	S	Cr	Ni	Mo	V	Nb	Co
C-1015 mild steel stud	0.17	0.55		0.009	0.001	–	–	–	–	–	–
Grade 50CR base plate	0.011	1.39	0.45	0.021	0.004	11.6	0.35	0.25	0.023	0.002	–
316L stainless steel stud	0.025	1.630	0.34	0.035	0.001	16.70	10.00	2.03	–	–	0.32

– Not specified in supplier provided MTR.

### Fabrication of steel shear studs

**Fig. 1:**
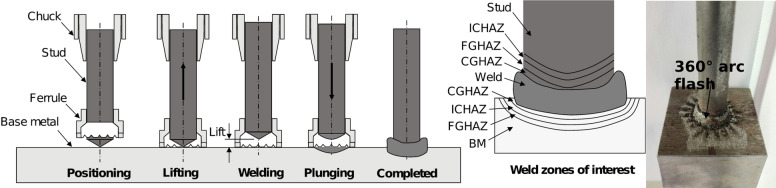
Schematic of a typical arc stud welding process and the formation of various local microstructural zones in the arc stud welded joint. Source: FHWA.

Welded-steel shear stud samples were prepared using recommended arc stud weld parameters (i.e., amperage: 1800 amps, time: 1.0 s, plunge length: 1/8 in.). Prior to the welding of studs on the base plate, the plates were thoroughly cleaned of any debris and oil. A schematic summarizing the arc stud welding process is provided in Fig. 1. All studs were welded to base plates in a flat position. The welded-steel shear studs were visually inspected for weld appearance and consistency. Specifically, the weld flash around the stud base was inspected for consistency and uniformity. A typical welded stud depicting the weld flash region is shown in Fig. 1. Stud welding follows a prequalified process, as per AWS D1.5 9.6, wherein a subset of welds gets qualified by satisfying a visual inspection and a destructive bending test^42^. During visual inspection, the stud flash is checked, ensuring that it covers the entire periphery. Destructive testing includes bending the unwelded end of the stud by 90 degrees by using a hammer. Once those requirements have been satisfied, no further verification is needed as long as the stud diameter and the weld process parameters remain unchanged^2^.

**Fig. 2:**
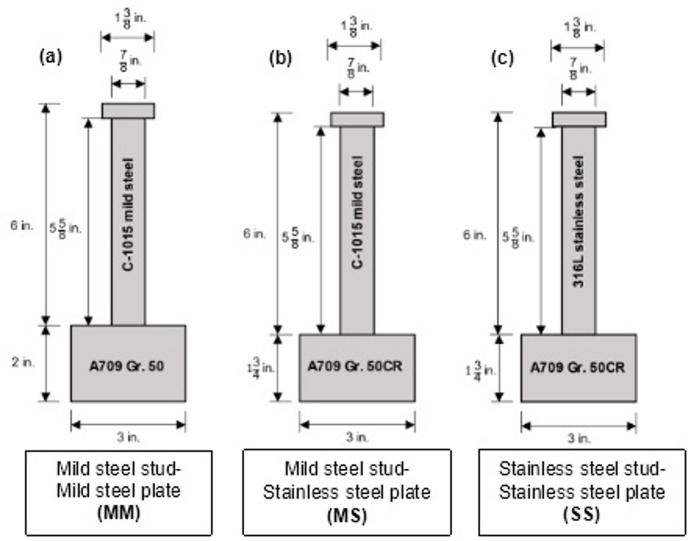
Geometric details of Stud–plate assemblies tested in this study: (**a**) mild carbon steel stud welded to Grade 50 plate steel plate (MM), (**b**) mild carbon steel stud welded to Grade 50CR steel plate (MS), and (**c**) 316L stainless steel stud welded to Grade 50CR steel plate. Source: FHWA.

In total, 30 double shear specimens were fabricated. The samples were equally divided into 3 groups of 10: Group MM (mild–mild) consisted of specimens with Grade C-1015 mild steel shear studs welded to either side of a 2-inch × 3-inch × 3-inch Grade 50 base plate; Group MS (mild–stainless) consisted of specimens with Grade C-1015 mild steel shear studs welded to either side of a 1¾-inch × 3-inch × 3-inch A1010 stainless steel base plate; and Group SS (stainless–stainless) consisted of specimens with 316L stainless steel studs welded to either side of a 1¾-inch × 3-inch × 3-inch A1010 stainless steel base plate. All three specimen groups were welded under identical process conditions. A geometric schematic of the specimens is provided in Fig. 2. The thickness of each base plate is within the specifications of the *PCI Design Handbook*, which requires a minimum base plate thickness of one-half of stud diameter^43^. Figure 2 shows that all studs have 7/8-inch diameters and are 6 inches long.

### Mechanical tests

Mechanical performance of the mild steel and stainless steel shear stud samples was evaluated by conducting double shear tests and uniaxial tension tests (i.e., pullout tests). For the 10 specimens in each of the material groups, 5 steel shear stud specimens were tested under double shear loading, and 5 specimens were subjected to uniaxial tension tests.

#### Shear strength tests

**Fig. 3:**
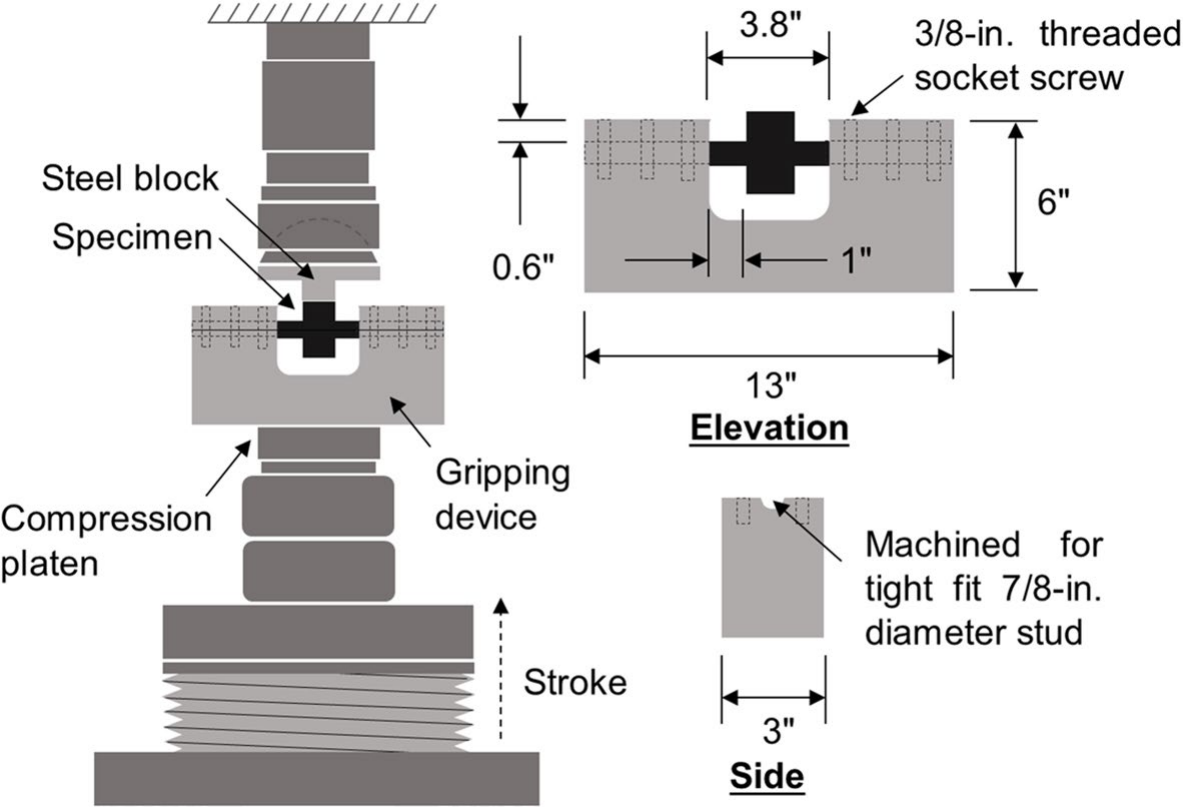
Schematic of shear test setup in universal testing machine. Source: FHWA.

To conduct shear tests, a custom shear test fixture was designed and machined. Figure 3 shows the test setup and a custom-made fixture for shear loading. The geometric details of the custom-made fixture are provided in Fig. 3. Figure 3 shows that the test setup essentially resembles a double shear test setup. The gripping device was machined to hold the samples rigidly, with an approximately 1-inch shear span. The welded shear stud specimens were seated in the gripping device such that both studs were clamped in the device’s grooves, and load was applied on the base plate to transfer a shearing force to the weld joints. Fixtures ensured proper alignment and minimized eccentric loading. Shear tests were performed on a 300-kip universal testing machine in displacement-control mode at a constant rate of 0.06 in/min (1.52 mm/min).

#### Uniaxial tension tests

**Fig. 4:**
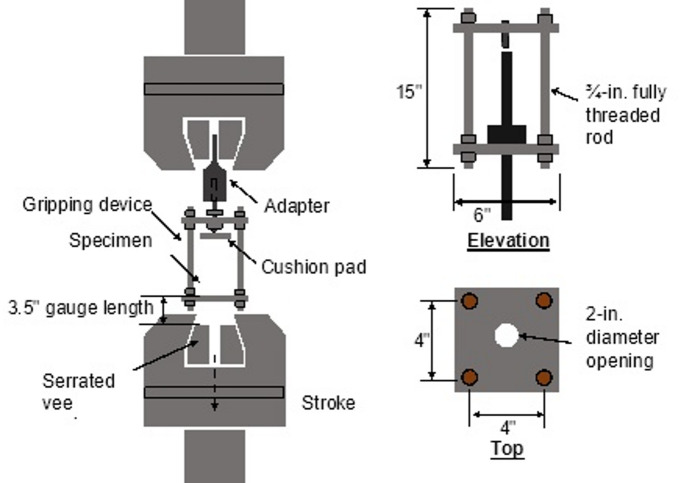
Schematic of tension test setup. Source: FHWA.

A custom gripping device was used to perform uniaxial tension tests on the shear stud specimens while minimizing bending. The test setup and custom gripping device are shown in Fig. 4. As shown in Fig. 4, the fixture comprises two 3/4-in. (19.05 mm) thick steel plates joined at the corners by four fully threaded 3/4-in. (19.05 mm) rods; each plate has a central 1.5-in. (38.1 mm) diameter bore to provide a clear axial loading path. The stud base plate rests on the lower plate and is clamped in a serrated V-wedge; the upper plate interfaces with a machined adapter that is held in the flat wedges of the universal testing machine. The stud under test is oriented vertically with a nominal 3-in. (76 mm) clear span between the clamping points; the top of the stud is free, and load is applied axially through the adapter and plates to the serrated wedge. This arrangement ensures concentric tensile loading and minimizes eccentricity and bending. Tests were conducted in displacement-control on a 100-kip (445 kN) universal testing machine at a constant crosshead rate of 0.2 in/min (5 mm/min) using guidance from ASTM E8. Displacement was measured by the machine. Five tension tests were performed for each stud group.

### Microstructure evaluation

Optical microscopy was used to characterize the local microstructure and metallurgical phases in the welded specimens. Microstructure examination was also used to assess the quality of the weld. Particularly, microstructure analysis was conducted in the following zones: (1) ferrite-pearlite matrix in the stud steel (unaltered microstructure); (2) stud heat-affected zone (HAZ), which consists of inter-critical HAZ (ICHAZ), fine-grain HAZ (FGHAZ), and CGHAZ; (3) weld metal, also referred to as “cast zone”; (4) base metal HAZ, which consists of ICHAZ, FGHAZ, CGHAZ; and (5) base metal (unaltered microstructure) to inquire as to the presence of specific metallurgical phases that formed due to the heating and cooling processes during welding.

Metallographic specimens were prepared by sectioning the welded shear stud specimens along the center to reveal the weld joint cross section. Metallographic specimens were extracted using a high-speed precision cutter with an appropriate cutting fluid to ensure the weld cross sections experience no additional heating or cooling during the cutting process. Metallographic specimens were subsequently prepared using standard metallographic specimen preparation procedures, as described in detail by Sajid et al.^44^ and Naik et al.^45^.

Different types of etchants were used to reveal the microstructure corresponding to each stud group. Two-percent nital etchant (nitric acid in alcohol)^46,47^ was used to reveal the microstructure and metallurgical phases in the MM specimens. For MS specimens, the surfaces were subjected to 2% nital etchant, which revealed the ferritic–pearlitic–martensitic structures in the stud and weld zone. The revealing of the structures was followed by etching with waterless Kalling’s No. 2 reagent^46,47^ to reveal grain structure in the ASTM A1010 base plate region. For SS specimens, Kalling’s No. 2 reagent was again used to reveal grain structure in the A1010 base plate and weld region, whereas Marble’s reagent^46,47^ was used to reveal the microstructure in the 316L stud and HAZ. Optical micrographs of the etched-steel specimens were then obtained for each steel shear stud group (MM, MS, and SS) at different magnification levels across the weld region corresponding to each zone of interest.

### Weld zone microhardness

The microstructure and metallurgical evolutions of the weld zones are further characterized through microindentation hardness transverses. Vickers indentations were performed adhering to ASTM E92^48^ and using a 500-gf indent (HV0.5) with a 13-sec dwell. To characterize the weld microhardness, three stud weld profiles—two edge profiles and one centerline profile—were defined across the weld zone of a typical metallographic specimen; the profiles span all regions of interest, as illustrated in Fig. 1. Indent spacing was 0.015 in (0.38 mm), in compliance with ASTM E92, with a minimum of three indents taken within each defined region.

Vickers hardness data were acquired along the profiles for each representative stud group in the following sequence: (1) unaltered stud steel; (2) stud HAZ, including ICHAZ, FGHAZ, and CGHAZ; (3) weld metal; (4) base metal HAZ, comprising ICHAZ, FGHAZ, and CGHAZ; and (5) base metal (unaltered microstructure). Those regions of interest encompass areas in which new or brittle metallurgical phases may have formed. The microhardness measurements thus provide direct evidence of any brittle phase formation that could lead to premature failure in weld joints, typically occurring at a probabilistic indicator threshold of 350 HV0.5. The microhardness data obtained from the three weld profiles for each of these regions was further averaged and reported as microhardness of the specific stud group.

## Results and discussions

This section describes results obtained from the experiments conducted on the welded shear stud specimens. The results include load-deflection curves for the double shear and uniaxial tension tests, discussion of failure modes in the weld, microstructure evolution, and microhardness across the weld profile.

### Shear strength

**Fig. 5:**
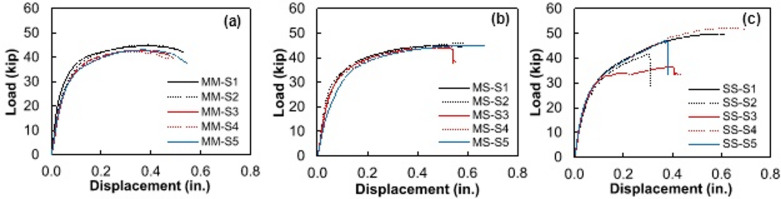
Load-displacement curves obtained from double shear tests on the welded stud assemblies: (**a**) Group MM shear studs, (**b**) Group MS shear studs, and (**c**) Group SS shear studs. Source: FHWA.

Shear strength of the welded shear stud, as defined in this study, refers to the maximum shear load experienced by the shear stud assembly (weld joint) before shear fracture. The shear strength was determined by conducting a double shear test on the welded shear studs, adhering to the procedure specified in “Shear strength tests”. The resulting load-displacement curves from the shear tests are presented in Fig. 5a through Fig. 5c.

**Fig. 6:**
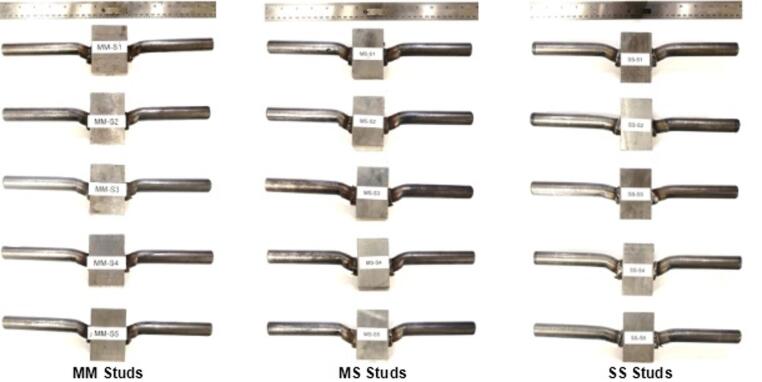
Photos of fractured welded steel shear studs subjected to shear tests. Source: FHWA.

**Fig. 7:**
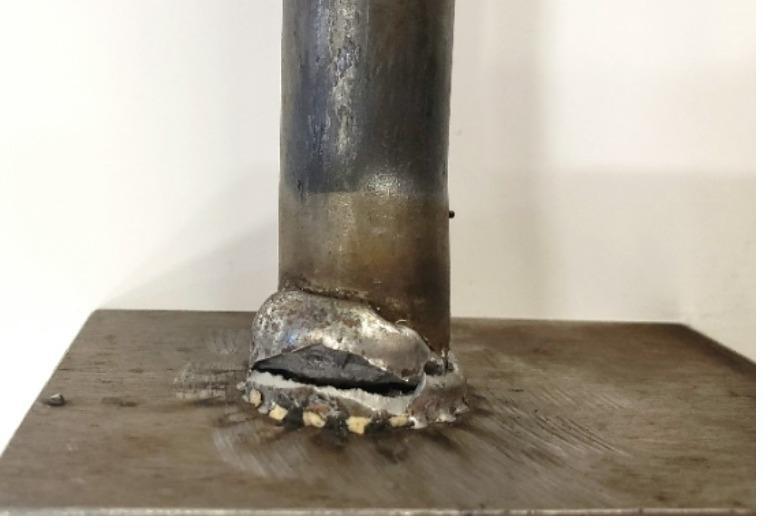
Weld fracture in SS-S3 sample during double shear test. Source: FHWA.

All three groups of shear specimens exhibited similar elastic responses, as expected for A36 and 316 L, which have comparable yield strengths, as shown in Fig. 5. The trend followed into the plastic loading region, with slightly higher ductility observed in the 316 L studs. Photos of tested steel shear studs are provided in Fig. 6. Specimen SS-S3 displayed fracture in the weld metal near the stud, as shown in Fig. 7. The premature failure occurred due to lack of soundness in the weld—likely due to the heterogeneity of the local weld microstructure or the potential presence of weld defects. These observed failure modes are discussed in the context of local weld-zone microstructural features and microhardness distributions in the subsequent section. Visual inspection of the tested specimens showed that the shear fracture occurred mostly in the weld joint, encompassing the HAZ and weld cast zone. Therefore, the results are excluded as outliers, and the welding parameters should be revisited. Similarly, specimens SS-S2 and SS-S5 experienced, to a lesser extent, premature failure due to lack of fusion between the weld metal and the stud. Micrographs in later sections of this paper show that good fusion for the 316L to 50CR is achievable; however, additional fine tuning of the dwell to amperage may improve the soundness of the weld along the circumference of the stud. Data from specimens SS-S2 and SS-S5 were included in the average response comparison.

Average maximum shear load capacity for each group of welded shear studs was nearly the same: MM: 43.48 kip; MS: 44.93 kip; and SS: 47.64 kip. Average shearing displacements for the MM and SS shear stud groups were relatively similar, recorded at 0.51 inch and 0.50 inch, respectively, while the MS shear stud group experienced an average displacement of 0.59 inch.

### Tensile strength tests

**Fig. 8:**
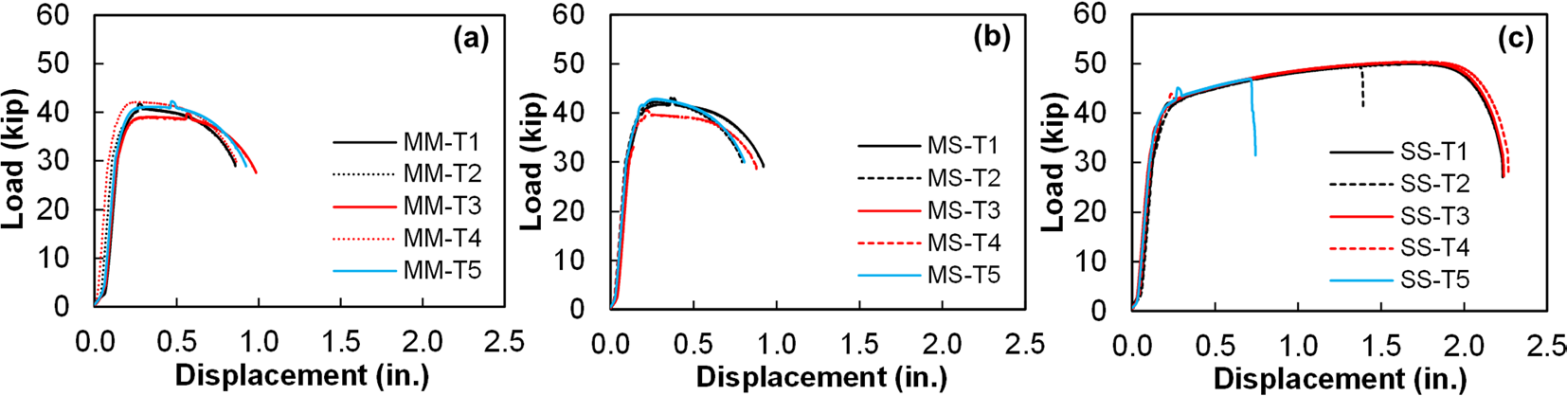
Load-displacement curves obtained from uniaxial tension tests on welded steel shear studs: (**a**) Group MM shear studs, (**b**) Group MS shear studs, and (**c**) Group SS shear studs. Source: FHWA.

Tensile strength of welded shear studs, as defined in this study, refers to the maximum tensile load a single stud and weld joint experiences before tensile failure. Tensile strength was determined by conducting uniaxial tension test on the welded shear studs, adhering to the procedure specified in “Uniaxial tension tests”. The resulting load-displacement curves of individual shear studs in each stud group—obtained from tension tests—are shown in Fig. 8a through Fig. 8c.

**Fig. 9:**
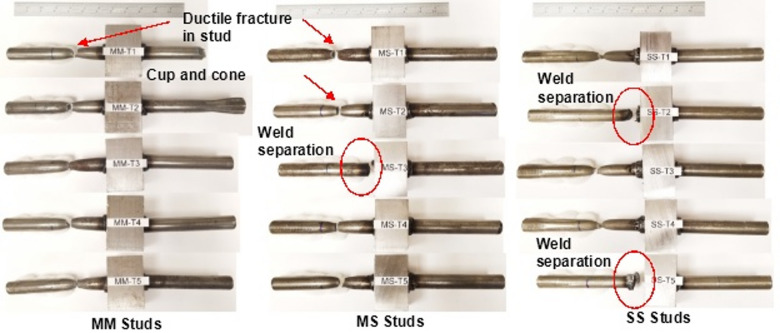
Tensile failure mode and fracture location in welded steel shear stud specimens; weld joint fractures are circled in red. Source: FHWA.

**Fig. 10:**
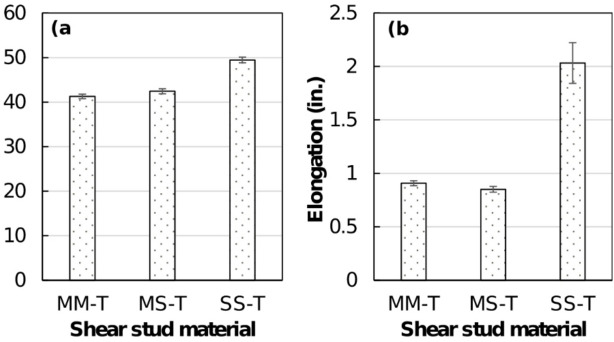
(**a**) Average maximum tension (ultimate load), and (**b**) average maximum elongation at fracture, for welded steel shear studs.

As shown in Fig. 8, the tensile load-displacement curves of the shear studs exhibit an initial elastic region followed by an extended plastic region. A well-defined ultimate load point and a necking region are evident in all the shear stud specimens. Notably, SS welded shear studs exhibited a more extended necking region compared with the MM and MS shear studs, as shown in Figs. 8 and 9, and quantified in Fig. 10. In most cases, typical ductile cup-and-cone type fracture occurred in the stud region, with the exception of one specimen in the MS group (MS-T3, where the stud separated from the weld) and two specimens in the SS group. In those cases, weld separation was observed. Figure 9 presents photographs of the tension-tested shear studs from each group, clearly showing necking in the studs.

For quantitative comparison, peak tensile (ultimate) loads and elongation at maximum load were calculated from load–crosshead displacement traces for each stud group and the average results are presented with standard-deviation error bars in Fig. 10a,b. Average maximum tensile load was comparable for the MM and MS shear studs, recorded at 41.28 kips and 42.44 kips, respectively. However, average maximum tensile load for the SS shear studs was 49.45 kips, which is 20% higher than that of MM shear studs and 15% higher than that of MS shear studs (see Fig. 10a). That higher tensile load capacity in SS shear studs can be attributed to a more pronounced strain hardening of the stainless steel studs compared with the mild steel studs, which contributed to the enhanced tensile capacity of the welded shear studs. For SS studs, two specimens failed at the weld joint, suggesting that the weld joint was weaker than the stud itself, which could be due to weld quality issues or the formation of brittle, weaker secondary phases in the weld joint region. To assess local weld microstructural properties, microstructure and hardness tests were conducted, and the following section discusses the results.

The maximum stud elongation at fracture—defined as the actuator (crosshead) displacement measured at the instant of tensile fracture—is presented in Fig. 10b for each stud group. MM specimens showed an average fracture elongation of 0.91 in (23.1 mm), while MS and SS groups exhibited mean elongations of 0.71 in (18.0 mm) and 1.77 in (45.0 mm), respectively. Thus, SS studs reached nearly twice the fracture elongation of MM studs and about 1.5 times that of MS studs. The greater elongation of the SS group is consistent with the higher ductility and strain-hardening capacity of 316L stainless steel and indicates that the tensile performance of the welded stud assemblies is largely governed by the stud material properties. Although two SS specimens fractured at the weld interface, those failures occurred at axial displacements equal to or exceeding the fracture displacements of the mild-steel studs, demonstrating that 316L stainless steel stud connections achieve comparable or superior ductility relative to conventional mild-steel studs.

Weld size and effective fusion area can influence whether a stud assembly fails in the stud shank or in the weld/HAZ. The load-carrying capacity of the stud–plate assembly is governed by the stud cross-sectional capacity and the effective weld and fusion area, as well as the local metallurgical strength and toughness of the weld metal and heat-affected zone (HAZ), which are influenced by fusion geometry, weld metal chemistry, and HAZ microstructure. Smaller weld size reduces the welded cross-section and increases the likelihood of weld/HAZ failure, whereas increasing fusion area shifts failure toward the stud. Achievable weld geometry is constrained by the ferrule and stud dimensions specified by the manufacturer. In this study the weld collar and fusion depth were established per AWS D1.5 and produced full-perimeter flash; nevertheless, some weld quality issue or localized high-hardness martensitic/M–A regions in some welds produced brittle zones likely caused weld/HAZ failures in a few specimens. A reduction in effective fusion area would proportionally lower weld capacity and could shift failure from ductile stud rupture to weld/HAZ separation, especially in dissimilar joints.

### Microstructure analysis

To examine local weld metallurgy and material heterogeneities within the weld HAZ and their influence on the mechanical behavior of welded shear studs, microstructural images were acquired for representative steel shear studs by adhering to the procedure specified in Sect. 2.4. Images were taken at close intervals and subsequently stitched together to create a composite photograph representing the entire weld profile. Additionally, microstructural images were obtained across the entire region of interest—including the stud, stud HAZ, weld metal, base metal HAZ, and base metal—for each group. Each shear stud group’s local weld microstructural features are discussed in the following subsections, with the aid of those images.

#### Mild steel stud on mild steel plate (MM)

**Fig. 11:**
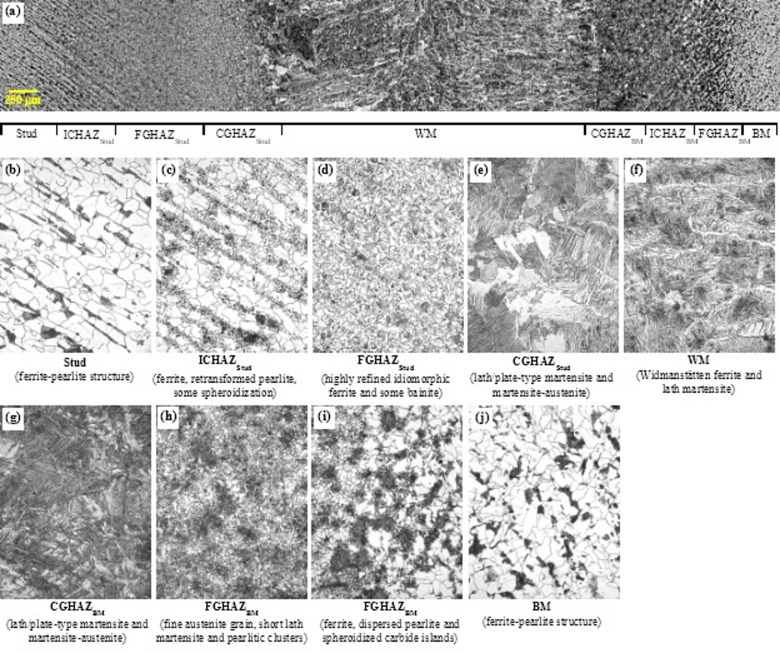
Microstructural characterization of the arc-stud welded shear-stud assembly (2% Nital etch). (**a**) Composite mosaic showing the full weld profile: stud shank, stud HAZ (ICHAZ, FGHAZ, CGHAZ), weld metal (WM), base-metal HAZ (CGHAZ, FGHAZ, ICHAZ), and unaltered base metal. Higher-magnification micrographs detail each zone: (**b**) Stud (ferrite–pearlite microstructure of low-alloy carbon steel), (**c**) Stud ICHAZ (polygonal ferrite with retransformed pearlite and isolated spheroidized carbides), (**d**) Stud FGHAZ (highly refined idiomorphic ferrite grains with upper-bainitic constituents), (**e**) Stud CGHAZ (coarse prior-austenite domains transformed into lath/plate-type martensite and martensite–austenite (M–A) microconstituents), (**f**) Weld metal (directional Widmanstätten ferrite plates and interspersed lath martensite), (**g**) Base-metal CGHAZ (lath/plate-type martensite and M–A microconstituents), (**h**) Base-metal FGHAZ (fine prior-austenite grains with short lath martensite and pearlitic clusters), (**i**) Base-metal ICHAZ (ferritic matrix with dispersed pearlite remnants and spheroidized carbide islands), and (**j**) Unaltered base metal (ferrite–pearlite microstructure). Source: FHWA.

A composite microstructural photo of the MM weld is provided in Fig. 10. The individual regions of interest in the weld profile—including the stud, stud HAZ (ICHAZ, FGHAZ, CGHAZ), weld metal, base metal HAZ (ICHAZ, FGHAZ, CGHAZ), and base metal—for the representative MM weld are annotated on the composite microstructural photo. Moreover, the microstructural photos of these individual zones are obtained at 40× magnification and are provided in Fig. 11. As seen in Fig. 11b, the unaltered stud and base metal exhibit a well-defined ferrite–pearlite morphology with light ferrite matrix and darker pearlite colonies. Phase and region assignments in the images are based on standard optical-metallographic criteria (2% Nital etch contrast and characteristic morphology) and by comparison with established literature for similar steels and heating-cooling conditions. The light grains show the ferrite phase, whereas the dark grains represent the pearlite colonies. The stud steel represents the unaltered microstructure, which is followed by the stud HAZ, which comprises ICHAZ, FGHAZ, and CGHAZ. Each of the three regions of the HAZ has a distinct morphology dictated by heat input, resulting in complex thermal cycles and cooling rate.

As observed in Fig. 11c, the ICHAZ microstructure depicts a partially transformed region, which consists mostly of polygonal ferrite with retransformed pearlite and occasional spheroidization—‍a phenomenon wherein the cementite layers in the pearlite structure progressively break up and shrink into spherelike structures and thus exist as spherelike structures in the ferrite matrix. ICHAZ temperatures can reach from upper critical temperature (A_3_, 910 ℃) to eutectoid temperature (A_1_, 723 ℃), and thus, partial pearlite grains partially transform to austenite. Upon cooling, some austenite can transform to martensite and exist in the form of small bands embedded in the softer, ferrite matrix.

The FGHAZ, also referred to as “grain-refined HAZ,” is characterized by the presence of highly refined idiomorphic ferrite granular structure and upper bainite (Fig. 11d), both of which are typically observed in the low-alloy steel FGHAZ microstructure^49–51^. The FGHAZ is typically exposed to peak temperatures ranging from upper-critical temperature (A_3_, 910 ℃) and austenite-grain-coarsening temperature (< 1,100 ℃). When low-alloy steels become exposed to such temperatures, ferrite (α-iron) grains completely transform to austenite (γ-iron) grains, which start to grow slowly. However, temperatures may not be high enough to completely dissolve carbides and other additives such as niobium or vanadium. Hence, the austenite grain boundaries are pinned by these additives, and thus their grain growth is restricted^44^. Abrupt cooling from this stage results in a fine-grained ferrite microstructure with upper bainite and some dispersion of martensite–austenite microphases^50^. The microstructural phases in the FGHAZ can have considerable impact on the hardness and toughness properties of the HAZ and the resulting weld, as discussed in the “Microhardness” section.

The CGHAZ microstructure, which refers to the grain-coarsened region of the stud HAZ, is typically subjected to temperatures above the grain-coarsening temperature of 1100 ℃ but below the melting temperature of the weld (1520 ℃)^52^. As previously discussed, ferrite grains completely transform to the austenite phase above the upper-critical temperature. When the temperature exceeds the grain-coarsening temperature, the carbides and other additives dissolve, and austenite grain growth occurs at a rapid pace, which leads to bigger austenite grains. The rate of austenite grain growth at this point depends on weld heat input. Upon rapid cooling, the large austenite grains retransform into an intergranular structure dominated by the presence of acicular structures—mostly lath martensite as shown by needlelike features in Fig. 11e and, possibly, some upper bainite. Arc stud welding delivers high instantaneous power at the stud–plate junction for a very short period; consequently, the heat input is highly localized (narrow HAZ) but produces steep thermal gradients and rapid cooling that can promote martensitic formation and local hardness peaks in the CGHAZ. The martensite phase is known to impart brittleness^53^ and reduced toughness of the local microstructure, which can ultimately lead to local brittle zones in the weldment^54,55^, as also observed in some shear test specimens. From a structural perspective, the presence of local brittle zones in the CGHAZ is of practical concern for steel bridges, as such regions are widely associated with reduced local fracture toughness and may increase susceptibility to brittle response under severe loading conditions or adverse environmental exposure.

The weld microstructure is formed by fusing portions of the stud and the base plate and is thus affected by the elemental composition and microstructural transformation of both the stud and the base plate steel. Grain growth in weld microstructure varies due to temperature gradients, and hence, the microstructure near the edge of the fusion line is slightly different from that at the center of the weld. Figure 11f shows that the weld microstructure has a typical dendritic structure morphology and consists primarily of directional ferrite grains with various secondary phases such as Widmanstätten ferrite (shown as sharp edges along with prior austenite grains)^50,56^, upper bainite, and acicular ferrite with a high aspect ratio^50,56^. Between the interspaces of the Widmanstätten ferrite, martensite phases are also present—and later confirmed by high hardness values. The weld metal is heterogeneous at the microscale—alternating lath packets, acicular/ Widmanstätten plates, and darker islands—which implies local variation in composition, cooling rate, and transformation paths across the weld. Such a microstructure typically produces locally elevated hardness in lath/martensitic regions and reduced toughness where M–A islands or brittle constituents concentrate. The observed morphology therefore explains the localized hardness peaks reported in the microhardness traverses and identifies likely microstructural sites for crack initiation or reduced fracture resistance under load.

The CGHAZ corresponding to the base metal HAZ has a morphology similar to that observed in the stud CGHAZ (Fig. 11g). However, the prior austenite grains in the base metal CGHAZ are considerably lower in size compared with the grains in the stud CGHAZ, which suggests that the base metal experienced slightly lower peak temperatures than the stud CGHAZ. This difference in grain size can be attributed to variation in thermal cycles during welding, where the stud CGHAZ is exposed to higher heat input and slower cooling rates, promoting greater austenite grain growth. Conversely, the base metal CGHAZ cools more rapidly, limiting grain coarsening. Nevertheless, the CGHAZ is dominated by the presence of lath martensite. The base metal FGHAZ shows a typical refined granular structure as that of stud FGHAZ. However, pearlitic structures are present, as shown by the dark clusters in Fig. 11h. Similarly, the base metal ICHAZ exhibits a relatively similar microstructural morphology of narrow bands of pearlitic structure along with occasional spheroidization, as observed in the stud ICHAZ (Fig. 11i). The unaltered base metal microstructure is composed predominantly of ferrite grains (shown by lighter grains in Fig. 11j) and pearlite colonies (shown by the dark areas). In summary, the unaltered microstructures in the stud and base metal are almost identical. Minor deviations are noticed between the base metal HAZ and the stud HAZ. Overall, these microstructural variations correlate with localized thermal histories influenced by weld parameters, dilution effects, and base metal properties, and they underpin the observed mechanical and hardness property differences discussed in subsequent sections.

#### Mild steel stud on stainless steel plate

**Fig. 12:**
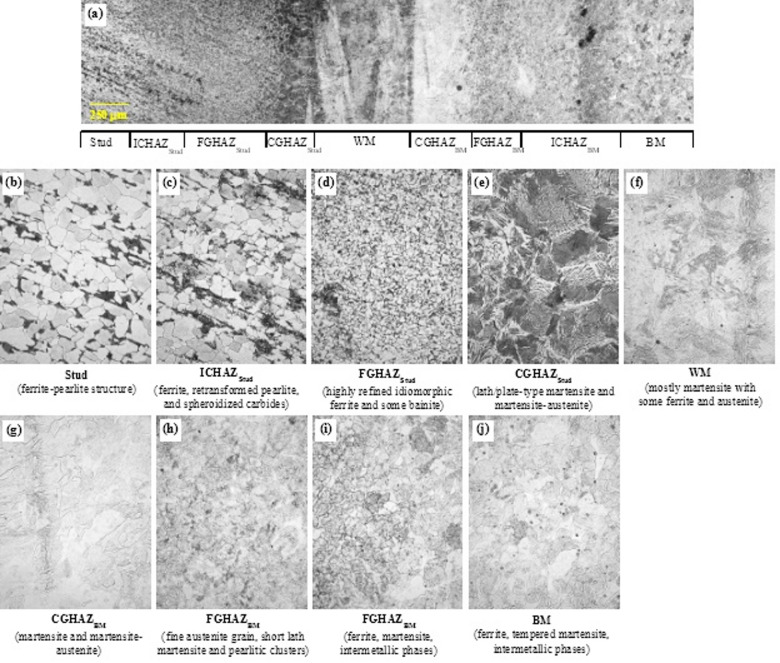
Microstructural characterization of the mild steel stud welded to Grade 50CR stainless steel plate (2% Nital etch followed by waterless Kalling’s No. 2 to expose grain structure in the Grade 50CR base plate). (**a**) Composite mosaic showing the full weld profile with annotated zones including stud, stud HAZ (ICHAZ, FGHAZ, CGHAZ), weld metal (WM), base-metal HAZ (CGHAZ, FGHAZ, ICHAZ), and unaltered base metal. Higher-magnification micrographs detail each zone: (**b**) Stud and stud HAZ showing typical ferrite–pearlite microstructure, (**c**) Stud ICHAZ with polygonal ferrite, retransformed pearlite, and spheroidized carbides, (**d**) Stud FGHAZ exhibiting fine-grained ferrite and upper bainite phases, (**e**) Stud CGHAZ dominated by coarse austenite grain transformation into lath martensite, (**f**) Weld metal microstructure marked by fusion of mild steel stud and stainless steel base plate, featuring heterogenous morphology with ferrite, dominant martensite, austenite, and intermetallic phases, (**g**) Weld microstructure morphology influenced by the Grade 50CR metallurgy, showing sporadic phase distribution, (**h**) Base metal FGHAZ composed of fine grains primarily of martensite, ferrite, austenite, and intermetallic compounds, (**i**) Base metal ICHAZ with morphology similar to unaltered Grade 50CR but with increased martensitic phase fraction, and (**j**) Unaltered Grade 50CR base metal showing elongated ferrite grains, tempered martensite bands, some retained austenite, and intermetallic phases. Source: FHWA.

A composite microstructural photo of the MS weld is provided in Fig. 12a, along with microstructural photos of individual regions at 40x magnification. Specimens were first etched with 2% Nital to reveal ferrite–pearlite–martensite in the stud and weld zones, then with waterless Kalling’s No. 2 to expose grain structure in the Grade 50CR base plate; phase identifications are based on optical etching contrast and characteristic morphology consistent with established metallurgical practice for weld-affected low-alloy steels. The unaltered stud steel and the stud HAZ microstructure are similar to those observed in the MM composite, comprising typical ferrite-pearlite structure (Fig. 12b). The ICHAZ microstructure consists of polygonal ferrite with retransformed pearlite and occasional spheroidization, wherein cementite layers break up into spherelike structures in the ferrite matrix (Fig. 12c). Temperatures in the ICHAZ range from upper-critical to eutectoid temperatures, which lead to partial transformation to austenite, some of which transforms into martensite upon cooling.

The FGHAZ is characterized by a fine-grained ferrite structure with upper bainite (Fig. 12d), typically observed in low-alloy steels exposed to peak temperatures from the upper-critical temperatures to austenite-grain-coarsening temperatures. The cooling from those temperatures results in a microstructure of refined ferrite, upper bainite, and dispersed martensite–austenite phases, which significantly affect the hardness and toughness of the weld. The CGHAZ experiences temperatures higher than the grain-coarsening temperature, whereby austenite grain growth occurs rapidly. Upon cooling, coarse prior-austenite grains transformed into a microstructure dominated by lath martensite (Fig. 12e). Such microstructures have been reported to exhibit increased brittleness and reduced toughness, which may have partially contributed to the failure modes discussed previously in the tensile and shear results sections.

The weld microstructure, as shown in Fig. 12f, is formed by fusing portions of the mild steel stud and Grade 50CR base plate and is thus affected by the elemental composition and microstructural transformation of those steels. Due to differences in the elemental compositions, to different principles of the solidification, and to the unique phase transformations of mild steels and stainless steels, grain morphology in the fusion zone is different in the vicinity of the stud CGHAZ versus base plate CGHAZ. Consequently, the weld microstructure in the MS group markedly differs from that observed in the MM specimens (Fig. 11), dominated by the influence of the Grade 50CR stainless steel base. Figure 12f demonstrate that weld microstructure heterogeneity arises from this mixing, presenting a sporadic morphology comprising ferrite, martensite, and retained austenite phases^57,58^, with martensite being dominant. Intermetallic phases are also present, identified as dark inclusions.

Unaltered Grade 50CR base metal microstructure consists primarily of elongated ferrite grains (shown by white regions in Fig. 12j) and bands of tempered martensite (shown by dark regions in Fig. 11) as well as some austenite grains^18,25,57^. Additionally, intermetallic phases—possibly containing aluminum, silicon, calcium, magnesium, and titanium—appear as dark marks in the base metal microstructure^57^. The Grade 50CR ICHAZ morphology closely resembles that of the unaltered base metal (Fig. 12i), though the increased density of darker regions suggests a slightly elevated martensite phase fraction, consistent with intercritical heating effects. The FGHAZ is characterized by a fine-grained mixture of martensite, ferrite, austenite grains, and intermetallic phases (Fig. 12h). The CGHAZ features larger grains composed of the same phase mixture but notably lacks detected intermetallic phases. These microstructural variations reflect the localized thermal history and dilution effects during welding, which influence mechanical properties and weld integrity, as discussed in subsequent sections.

#### Stainless steel stud on stainless steel plate

**Fig. 13:**
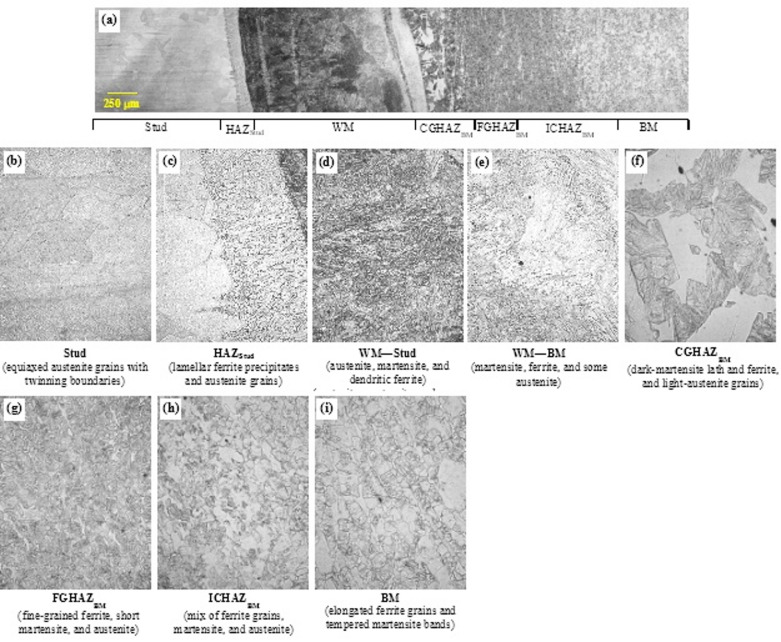
Microstructural characterization of the stainless-steel (SS) stud welded to Grade 50CR stainless-steel base plate, revealed using Marble’s reagent (316L stud/HAZ) and waterless Kalling’s No. 2 (Grade 50CR base/weld). (**a**) Composite mosaic of full weld profile showing stud, stud HAZ, weld metal (WM), and base-metal HAZ (ICHAZ, FGHAZ, CGHAZ), and unaltered base metal (BM). Higher-magnification micrographs detail each zone: (**b**) Stud-equiaxed austenite grains with twinning boundaries and carbides; (**c**) Stud HAZ-lamellar ferrite precipitates surrounding austenite grains; (**d**) WM (stud side)-austenite, martensite, and δ-ferrite as dendritic ferrite; (**e**) WM (base-metal side)-martensite, ferrite, and some austenite; (**f**) Base metal CGHAZ-dark martensitic laths and ferrite with light austenite grains; (**g**) base metal FGHAZ-fine-grained ferrite, short martensite, and austenite; (**h**) base metal ICHAZ-mixture of ferrite grains, martensite, and austenite; (**i**) BM-elongated ferrite grains, tempered martensite bands, some austenite, and intermetallic phases. Source: FHWA.

A composite microstructural photo of the stainless steel weld is provided in Fig. 13a, along with microstructural photos of individual regions at 40× magnification. For SS specimens, Kalling’s No. 2 reagent was used to reveal grain structure in the Grade 50CR base plate and weld region, whereas Marble’s reagent was used to reveal the grains in the 316L stud and HAZ. As shown in Fig. 13b, the unaltered 316L stud steel microstructure exhibits typical equiaxed austenite grains, with twinning boundaries and the presence of some carbides. A very narrow HAZ is formed in the 316L stud, contrary to the mild steel studs, in which clearly distinguishable intercritical and supercritical HAZs were present. The 316L stud HAZ is composed primarily of lamellar ferrite precipitates—as shown by lathy and skeletal dark lines in Fig. 13c—that surround austenite grains.

The weld microstructure near the 316 L fusion line is different from the fusion line on the Grade 50CR base metal side. From the Grade 50CR base metal side, the weld microstructure is most likely composed of martensite, ferrite, and austenite grains as well as intermetallic phases (Fig. 13e). That assumption is supported by a previous research investigation^57^. From the 316L stud side, the weld microstructure is more complex. It most likely consists of austenite, martensite, and some δ -ferrite in the form dendritic ferrite (Fig. 13d). These microstructure phases align well with the Schaeffler diagram, which is commonly used for predicting phase composition in the fusion zone. The microstructure corresponding to the weld and stud HAZ does not appear to show any sigma phase, which—from a corrosion resistance perspective—is typically a troubling phase.

The HAZ microstructural phases and morphologies toward the Grade 50CR base metal side were relatively similar to those observed in the MS specimens. The unaltered Grade 50CR microstructure is composed primarily of elongated ferrite grains and tempered martensite bands, along with some austenite grains (Fig. 13i). Additionally, dark markings in the figure indicate the presence of intermetallic phases. The Grade 50CR ICHAZ shares a structure similar to that of the base metal, but it shows a higher concentration of darker areas, implying an increased presence of martensite compared with the base metal (Fig. 13h). In contrast, the FGHAZ is characterized by a finer grain structure, consisting primarily of martensite, ferrite, and austenite, along with intermetallic phases (Fig. 13g. The CGHAZ, on the other hand, features larger grains and retains a similar phase composition but without any noticeable intermetallic phases (Fig. 13e). These morphologies have considerable impact on the microhardness of the weld profile, as discussed in the following section.

### Microhardness results

**Fig. 14:**
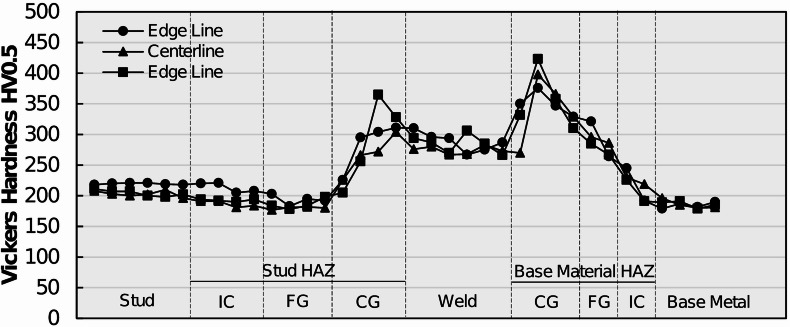
Microhardness readings across the arc stud welded joint profiles of a typical MM steel shear stud. Source: FHWA.

**Fig. 15:**
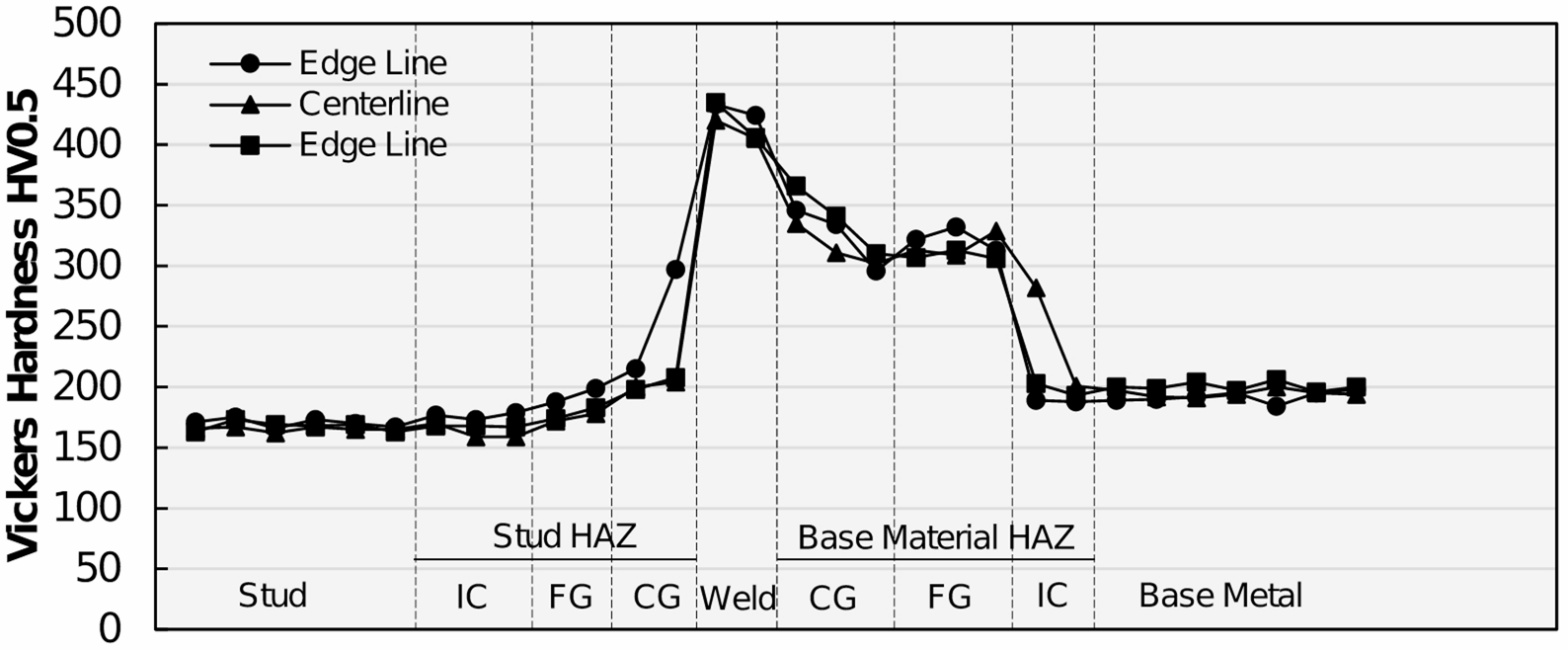
Microhardness readings across the arc stud welded joint profiles of a typical MS steel shear stud. Source: FHWA.

**Fig. 16:**
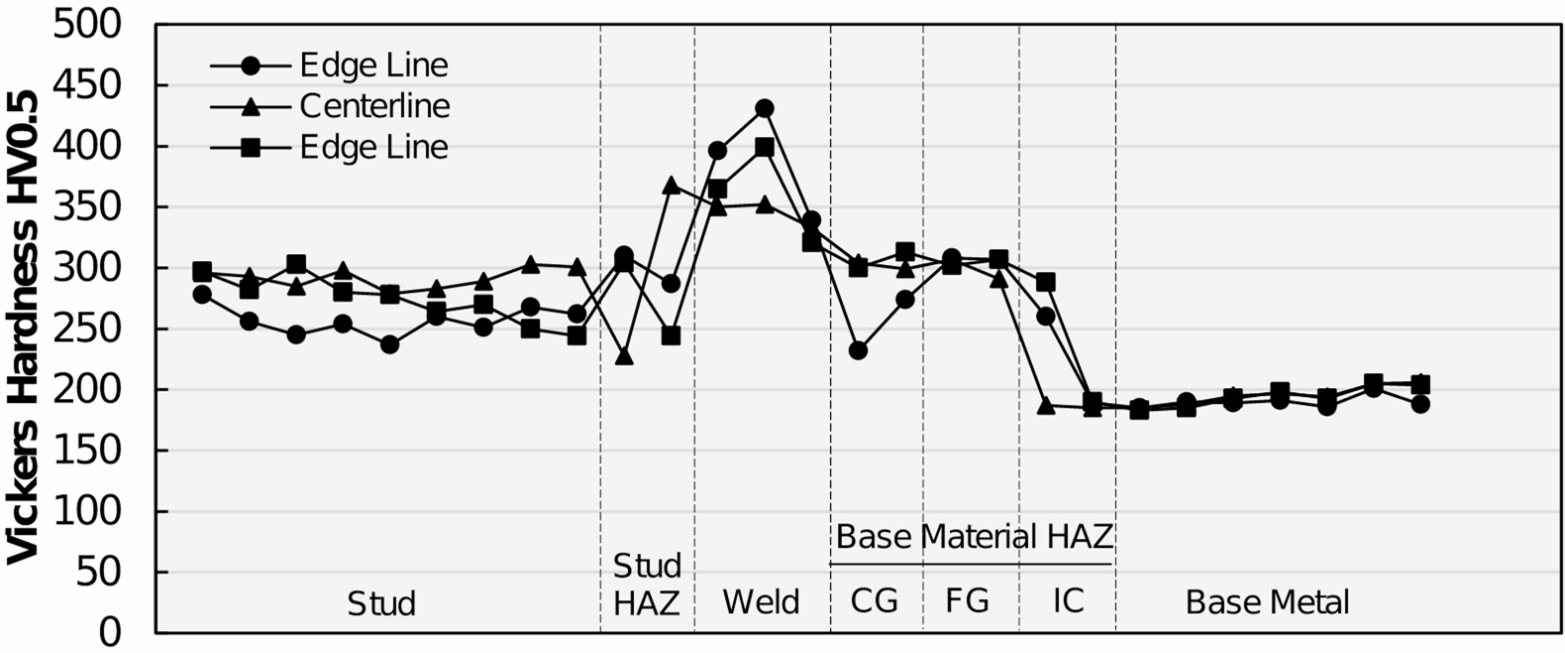
Microhardness readings across the arc stud welded joint profiles of a typical SS steel shear stud. Source: FHWA.

To characterize the local weld metallurgy and material heterogeneities’ arc stud welded joints by means of strength, Vickers microhardness indentation testing was conducted on representative shear studs from each group by adhering to the procedure specified in Sect. 2.5. Hardness transverses were performed on metallographic specimens by using a low-force test range (HV0.5) across three stud profiles: two edge profiles and one centerline profile. The profiles are meant to determine average microhardness as well as any difference in hardness behavior in the central and external regions of the welded joints. Representative Vickers hardness transverses for MM, MS, and SS specimens are plotted in Figs. 14 and 15, and Fig. 16, respectively.

As observed in Fig. 14, the MM specimens measured an average Vickers hardness of 209 HV0.5 in the unaltered stud metal. Vickers hardness remains relatively unchanged in the stud ICHAZ (198 HV0.5). However, as the profile gets traversed from ICHAZ toward the fusion line, the hardness values increase substantially. The average hardness value in the stud CGHAZ is recorded to be 280 HV0.5, with local spikes up to 365 HV0.5. The weld microstructure has an average hardness value of 282 HV0.5. That substantial increase in local weld microhardness can be attributed to the formation of the brittle martensite phase, as discussed in the preceding section. A similar trend in hardness values is noticed in the base metal HAZ and unaltered base metal. The base metal CGHAZ exhibited an average Vickers hardness of 349 HV0.5, with local spikes reaching as high as 425 HV0.5 due to the presence of the lath martensite phase. The average Vickers hardness value then reduces slightly in the base metal FGHAZ (316 HV0.5) and ICHAZ (209 HV0.5) while reducing to an average Vickers hardness of 196 HV0.5 in the base metal. These trends highlight the presence of localized brittle phases within the weld metal and HAZ, which may influence deformation compatibility and fracture behavior and help explain the weld- or HAZ-related failures observed in a limited number of tensile and shear tests.

The transverses along the edge lines and centerlines of a representative MS specimen are provided in Fig. 15. The mild steel stud exhibited an average Vickers hardness of 168 HV0.5. The hardness increased only nominally as the weld profile gets traversed from the stud to the stud FGHAZ. The stud ICHAZ and FGHAZ exhibited average Vickers hardnesses of 169 HV0.5 and 182 HV0.5, respectively. In the CGHAZ and weld regions, Vickers hardness increased substantially compared with hardness of the stud or the base metal. Average Vickers hardness in the stud CGHAZ was observed to be 220 HV0.5, with local spikes of 300 HV0.5. The weld microstructure exhibited an average microhardness of 410 HV0.5, which is more than two times the hardness of unaltered stud steel. That sharp increase in hardness in the weld zone can be attributed to the martensite-dominant weld microstructure as well as intermetallic and δ-ferrite. After reaching the maximum, Vickers hardness starts descending as the profile gets traversed toward the base metal HAZ. The base metal CGHAZ and FGHAZ exhibited average Vickers hardnesses of 324 HV0.5 and 309 HV0.5, respectively. The base metal ICHAZ had an average Vickers hardness of 209 HV0.5, which is similar to the Vickers hardness of 195 HV0.5 observed in the unaltered CR50 base metal.

Average Vickers hardness results of SS specimens are plotted in Fig. 16. The unaltered stud metal exhibited an average Vickers hardness of 274 HV0.5, which is typical for a 316L stainless steel. Average Vickers hardness in the stud HAZ was observed to be 290 HV0.5. The weld microstructure showed an average Vickers hardness of 365 HV0.5, which is substantially higher than the stud hardness. Moreover, the hardness of the edge lines was observed to be higher than the hardness of the weld centerline and could be attributed to the higher cooling rate at the exterior of the weld. The hardness values then decreased in the base metal CGHAZ, FGHAZ, and ICHAZ from the peak values observed in the weld microstructure. The average hardness in the base metal CGHAZ, FGHAZ, and ICHAZ were observed to be 287 HV0.5, 304 HV0.5, and 216 HV0.5, respectively. The unaltered base metal exhibited an average hardness of 193 HV0.5. That the peak hardness values were less pronounced for the SS specimens compared with MS specimens is evident.

**Fig. 17:**
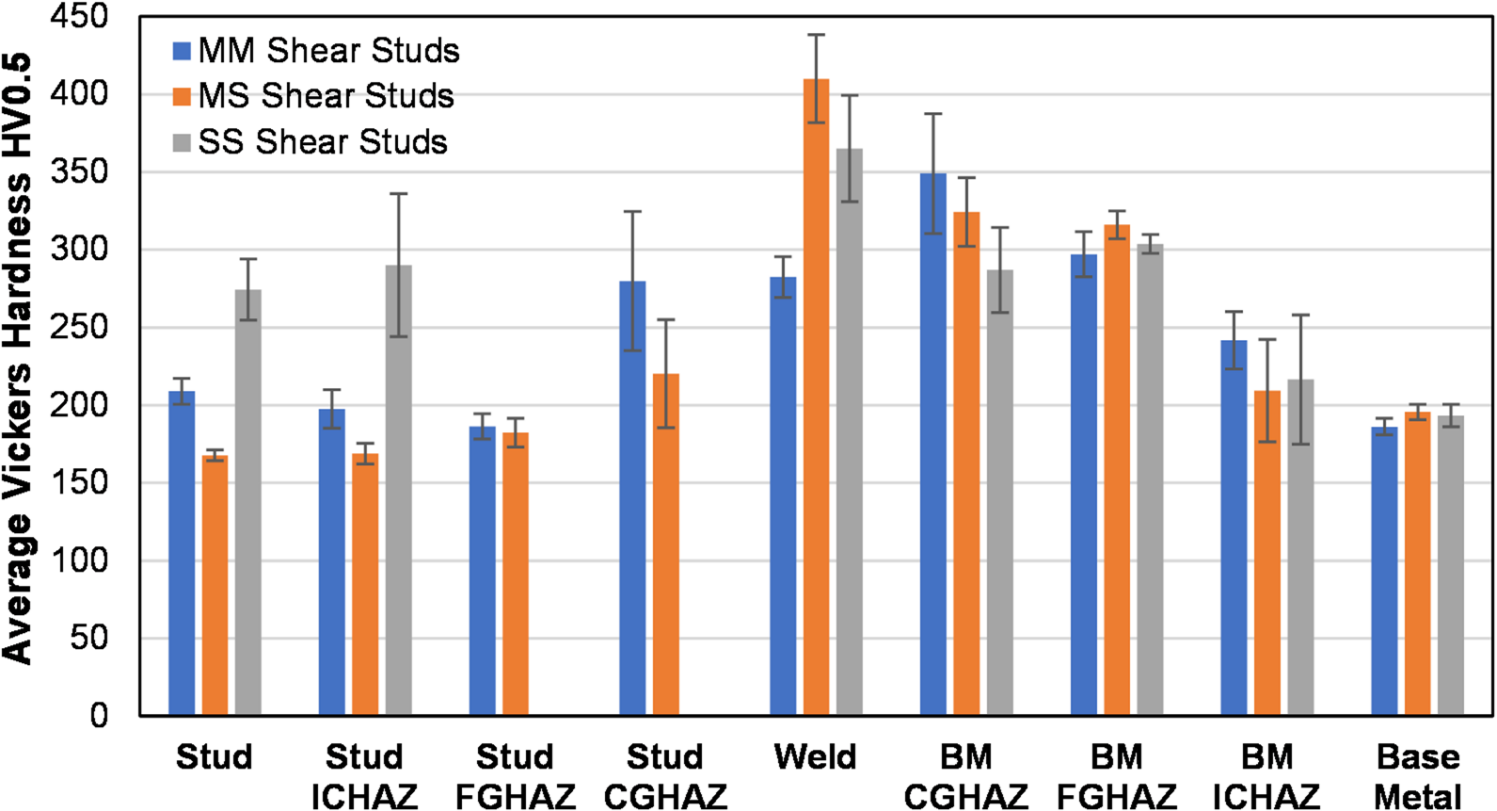
Average microhardness corresponding to different local weld zones in the MM, MS, and SS shear studs; the error bars represent one standard deviation. Source: FHWA.

The average microhardnesses of the individual regions of interest for each shear stud group are compared with error bar plots in Fig. 17. That the SS group has the highest average hardness in the stud region is evident—and expected for stainless steel. Interestingly, among the three groups, the MS group had the highest average microhardness in the weld microstructure, which suggests that the MS group developed a higher proportion of undesirable brittle phases in the weld microstructure, and which thus may lead to a local brittle zone and compromise the integrity of the welded joint. Such results demonstrate the potentially undesirable effects of material mismatches and complex weld metallurgy in such dissimilar welded joints. Localized elevated microhardness is of practical relevance for bridge welds, as such hardness gradients are commonly associated with brittle microstructural constituents and may indicate regions of reduced local toughness under demanding service conditions.

## Conclusions

The primary conclusions of this research are as follows:

1. The mechanical performance of welded shear studs in tension and shear was comparable across stainless-steel and mild-steel configurations, with 316L stainless steel studs demonstrating notably higher ductility.
2. Premature fracture within the weld occurred in some specimens, attributed to local weld heterogeneities, potential embrittlement, or weld defects. These fractures predominantly involved the weld joint, including the heat-affected and fusion zones.
3. The weld microstructure contained brittle phases such as martensite, upper bainite, and Widmanstätten ferrite. Vickers hardness measurements exceeding 400 HV0.5 support the presence of these hard phases. Optimization of welding parameters may reduce the formation of such phases and improve weld uniformity, potentially enhancing stud joint performance. Despite some premature failures, 316L stainless steel studs exhibited strength and ductility comparable to or surpassing those of mild-steel studs.
4. No sigma phase formation was detected within the weld and stud heat-affected zones. This finding aligns with established metallurgical expectations under the employed welding conditions and is significant as sigma phase can adversely affect corrosion resistance.
5. The unaltered base and stud metals exhibited characteristic microstructures for their grades. Grade 50CR base metal primarily consisted of elongated ferrite grains, tempered martensite bands, some austenite, and minor intermetallic phases. The adjacent heat-affected zone displayed a finer grain structure with an increased martensite fraction. Vickers hardness values in the heat-affected zones generally remained below 350 HV0.5, indicating satisfactory weldability of Grade 50CR steel.

This research was conducted primarily to deal with concerns about the weldability and performance of ASTM A709 Grade 50CR as a base metal. Although welding conventional mild-steel studs to stainless (Grade 50CR) base plates is generally feasible from a weldability standpoint, such dissimilar-metal joints can create galvanic-corrosion risks in service. The results show that 316L stainless steel shear studs welded to stainless base plates provide comparable or improved tensile and shear performance relative to mild studs while eliminating the dissimilar-metal galvanic concern, making stainless-on-stainless stud connections an attractive option where long-term corrosion resistance is a priority. While this study establishes the mechanical viability of austenitic 316L stainless steel shear studs welded to Grade 50CR stainless steel base plates, further investigations are warranted to comprehensively evaluate their long-term performance in bridge applications. Future investigation should also include a parametric weld-size/fusion-area study and quantify their influence on the failure behavior of shear stud and the joint.

## Data Availability

The authors declare that the data supporting the findings of this study are available within the paper. Should any raw data files be needed in another format they are available from the corresponding author upon reasonable request.
